# Symplasmic Isolation Contributes to Somatic Embryo Induction and Development in the Tree Fern *Cyathea delgadii* Sternb

**DOI:** 10.1093/pcp/pcaa058

**Published:** 2020-05-06

**Authors:** Małgorzata Grzyb, Justyna Wróbel-Marek, Ewa Kurczyńska, Mirosław Sobczak, Anna Mikuła

**Affiliations:** p1 Department of Conservative Plant Biology, Polish Academy of Sciences Botanical Garden–Center for Biological Diversity Conservation in Powsin, Prawdziwka 2, Warsaw 02-973, Poland; p2 Institute of Biology, Biotechnology and Environmental Protection, Faculty of Natural Sciences, University of Silesia in Katowice, Jagiellonska 28, 40-032 Katowice, Poland; p3 Institute of Biology, Department of Botany, Warsaw University of Life Sciences (SGGW), Nowoursynowska 159, Warsaw 02-787, Poland

**Keywords:** Cell wall, Fluorochromes, Somatic embryogenesis, Ultrastructure

## Abstract

In this report, we describe studies on symplasmic communication and cellular rearrangement during direct somatic embryogenesis (SE) in the tree fern *Cyathea delgadii*. We analyzed changes in the symplasmic transport of low-molecular-weight fluorochromes, such as 8-hydroxypyrene-1,3,6-trisulfonic acid, trisodium salt (HPTS) and fluorescein (delivered to cells as fluorescein diacetate, FDA), within stipe explants and somatic embryos originating from single epidermal cells and developing during 16-d long culture. Induction of SE is preceded by a restriction in fluorochrome distribution between certain explant cells. Microscopic analysis showed a series of cellular changes like a decrease in vacuole size, increase in vacuole numbers, and increased density of cytoplasm and deposition of electron-dense material in cell walls that may be related with embryogenic transition. In somatic embryos, the limited symplasmic communication between cells was observed first in linear tri-cellular embryos. Further development of the fern embryo was associated with the formation of symplasmic domains corresponding to the four segments of the plant body. Using symplasmic tracers, we provided evidence that the changes in plasmodesmata permeability are corelated with somatic-to-embryogenic transition and somatic embryo development.

## Introduction

Somatic embryogenesis (SE) is a process facilitating the development of structures resembling the zygotic embryos from somatic cells of a plant body, which undergo dedifferentiation events and an orderly arrangement of characteristic developmental stages. This phenomenon has been extensively studied at the levels of genes, proteins, metabolites and morphology, including analyses of relationships between the cell wall, plasma membrane and cytoskeleton (Fehér 2015). Elucidation of mechanisms leading to the formation of somatic embryos is difficult due to the different pathways of SE initiation, and the strikingly different and highly sophisticated conditions that can trigger this process (Radoeva and Weijers 2014, Fehér 2015). A unique experimental system of SE in the tree fern *Cyathea delgadii*, described for the first time in 2015 (Mikuła et al. 2015b), can be a helpful tool to understand various events that occur during the somatic-to-embryogenic transition (Mikuła et al. 2018). *Cyathea delgadii* belongs to a small group of plants in which the explant cells can be reprogrammed in vitro to form somatic embryos in the absence of exogenous growth regulators (Mikuła et al. 2015a). The pathway of somatic embryo initiation and development in *C. delgadii* explants depends on such artless factors as length and diameter of the explant-donor frond, type of explant, as well as physical and chemical factors of media and culture conditions (Mikuła et al. 2015a, Grzyb and Mikuła, 2019). One of the basic triggers inducing SE is the light conditions for donor plantlet growth (Mikuła et al. 2015a), which regulate the endogenous hormone contents and hormonal balance (Grzyb et al. 2017). However, the most interesting feature of this model seems to be the epidermal and unicellular origin of somatic embryos (Mikuła et al. 2015b). Studies on *C. delgadii* showed that the first cellular divisions leading to the formation of somatic embryos are different from those occurring during zygotic embryogenesis. The early stage of SE is characterized by several cell divisions perpendicular to the apical–basal axis of the stipe explant, and they lead to the formation of a linear embryo (Mikuła et al. 2015b). By contrast, the zygote divides into four daughter cells that initiate the four quarters of the zygotic embryo body of the fern (Johnson and Renzaglia 2008). The proliferation of zygotic initial cells depends on the orientation of the zygote in the archegonium and its position in relation to the gametophyte axis. The four quadrants will form root, leaf, foot and shoot apex of zygotic embryo (Johnson and Renzaglia 2008). Despite differences in the initiation of the somatic and zygotic embryos, later their development is similar and they are able to develop into functional sporophytes (Mikuła et al. 2015b, Mikuła et al. 2018).

Plasmodesmata (PD) are recognized as essential regulatory elements in the development of multicellular plants (Otero et al. 2016). They facilitate the communication and transport of materials, including signaling molecules, between plant cells (Zambryski 2004). The diameter and permeability of these symplasmic channels may be modified during cell/organ development or in response to external conditions and stimuli (Zambryski and Crawford 2000, Marzec and Kurczynska 2014). The functionality and role of PD was widely explored in terms of organ development (Ding et al. 1999, Marzec and Kurczynska 2014, Otero et al. 2016), floral transition (Ormenese et al. 2000), dormancy induction in apical buds (Jian et al. 1997), embryo development during zygotic embryogenesis (Kim and Zambryski 2005, Stadler et al. 2005, Wróbel-Marek et al. 2017) and androgenesis (Wrobel et al. 2011). Many studies on symplasmic communication were based on the transport of tracers such as low-molecular fluorochromes, fluorescent-labeled dextrans or green fluorescent protein (Crawford and Zambryski 2000, Kim et al. 2002, Wrobel et al. 2011, Marzec et al. 2014). Cell-to-cell transport of these substances allows comparing PD permeability for molecules of different size during developmental processes (Zambryski 2004). Although cell-to-cell communication is a commonly accepted key that switches development from vegetative growth into an embryogenic path, the issue is still poorly understood (Kurczynska et al. 2012). During the induction of SE, the symplasmic communication is most often studied on the level of cellular changes. Of these, the cytolocalization of glucans, pectin epitopes or callose deposition is most frequently considered in response to osmotic challenges (Puigderrajols et al. 2001, Verdeil et al. 2001, You et al. 2006, Grimault et al. 2007). The analyses of symplasmic communication during SE with the use of fluorescent labels were performed only for Arabidopsis and showed the restriction in cell-to-cell movement through PD between parts of explants engaged in SE and those that were not involved in somatic embryo formation (Kurczyńska et al. 2007, Kulinska-Lukaszek and Kurczynska 2012, Godel-Jedrychowska et al. 2020).

The involvement of PD in the regulation of cell differentiation and developmental patterns in ferns was first proposed many years ago, but it applies in particular to the gametophyte formation. It was shown that plasmolysis, which breaks down the PD connections, led to abnormal branching of filamentous fern gametophytes and to the re-differentiation of non-apical cells into apical type cells (Nakazawa 1963). This reaction was apparently caused by the interrupted transmission of information determining the normal pattern of development (Drake et al. 1978). Functional PD are required for the normal development of *Onoclea sensibilis* gametophyte (Tucker 1990). Disruption of symplasmic communication induces each cell of the gametophyte to become totipotent and to develop into an individual heart-shaped prothallus. Another example of the role of PD in fern development is provided by the discovery that symplasmic isolation of the apical cell of *Azolla* sp. root occurs by a reduction in the number of PD as a result of successive divisions (Robards and Lucas 1990). This ‘dilution’ of PD frequency is thought to control the determinate growth pattern of the *Azolla* sp. root (Robards and Lucas 1990) and the directions of apical cell divisions in the fern gametophyte (Holloway and Lantin 2002).

The aforementioned data encouraged us to ask whether the somatic-to-embryogenic transition in ferns is associated with changes in symplasmic communication. Such information would increase our knowledge of the role of PD from a physiological and evolutionary point of view that is presently lacking for fern SE. In this study, we analyzed the intercellular communication system that can cause epidermal cells of stipe explants to become embryogenic through a reduction in information exchange between explant cells. To that end, analyses of the distribution of fluorescent low-molecular-weight symplasmic tracers within the explants and early somatic embryos were undertaken to answer whether there is a correlation between changes in symplasmic communication and cell differentiation during fern SE.

## Results

### Fluorochrome distribution within initial explants

**Fig. 1:**
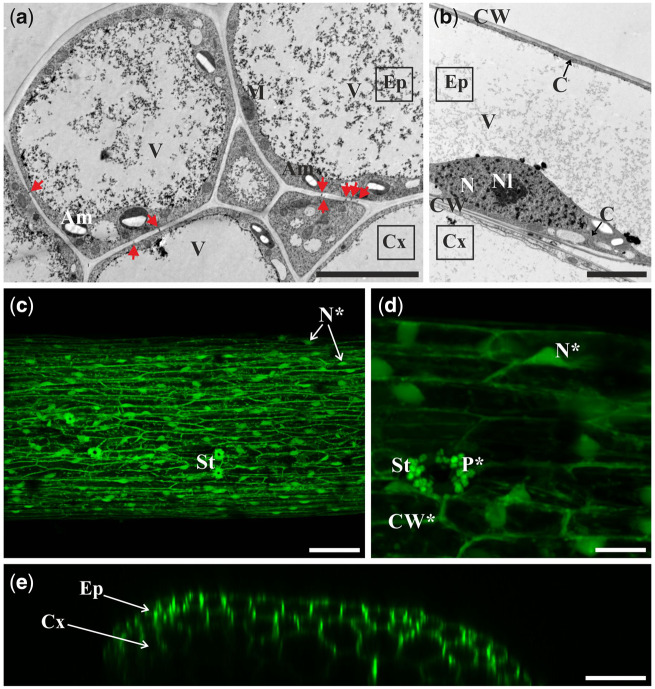
Microscopic analysis and symplasmic tracer distribution in the initial stipe explants. (a) Cross-section of highly vacuolated epidermal and cortical cells. (b) The epidermal cell contains thin layer of cytoplasm and nucleus located near the cell wall neighboring cortex (longitudinal section of explant). (c) HPTS accumulation (green) in the epidermal cells of the initial explant (top surface view of the middle part of the explant is shown). (d) HPTS accumulation (green) in the thin peripheral cytoplasm next to the cell wall (CW*), around the nuclei (N*) and around plastids (P*) of epidermal cells. (e) Orthogonal projection of the same explant as depicted in (d). (c–e) Explant incubated in aqueous solution of HPTS for 60 min. TEM (a, b) and CLSM (c–e) images. Bars = 5 µm (a, b), 100 µm (c), 25 µm (d) and 50 µm (e). Am, amyloplast; C, cytoplasm; CW, cell wall; Cx, cortex; Ep, epidermis; M, mitochondrion; N, nucleus; Nl, nucleolus; V, vacuole; St, stoma; red arrows, plasmodesmata.

Epidermal and cortical cells of stipe explants were highly vacuolated and contained well-developed central vacuoles (Fig. 1a, b). In epidermal cells, the nucleus was located near the cell wall adjacent to the cortex (Fig. 1b). A thin layer of cytoplasm was located against the cell wall (Fig. 1a) and surrounded the nucleus (Fig. 1b). PD were present between epidermal cells as well as between epidermal and cortical cells (Fig. 1a).

In the initial explant, 8-hydroxypyrene-1,3,6-trisulfonic acid (HPTS) was present within the epidermal cells (Fig. 1c–e) and in 2–3 cell layers of the cortex (Fig. 1e). Fluorochrome distribution in epidermal cells was uniform along the entire length of the explant (Fig. 1c). Inside the cell, fluorochrome was localized in the cytoplasm (layer present near the cell wall, around nucleus and plastids) and in cytoplasmic strands crossing the vacuole (Fig. 1d). Fluorochrome was also detected in plastids (Fig. 1d). A similar pattern of fluorochrome distribution was observed following fluorescein diacetate (FDA) treatment (data not shown). The results obtained indicate that, in the initial stage, the whole explant is a single continuous symplasmic domain, at least in relation to epidermis and few cell layers of cortex directly below it.

### Fluorochrome distribution within explants (6th day of culture)

**Fig. 2:**
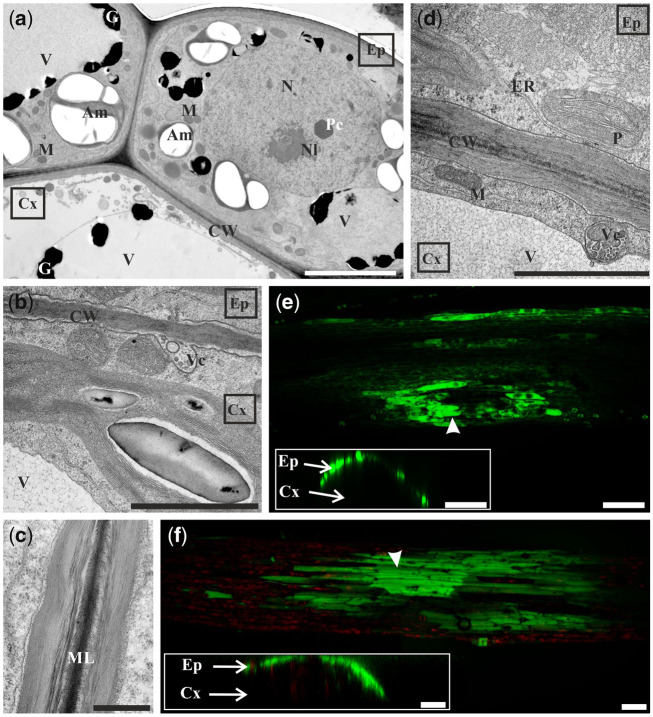
Microscopic analysis and symplasmic tracer distribution in 6-day-old explants. (a) Epidermal cells have dense cytoplasm, large nucleus, numerous mitochondria and amyloplasts and vacuoles with electron-dense granules. Cortical cell has electron-lucent cytoplasm (cross-section of explant). Ultrastructural details of the cell walls between epidermis and cortex (b, d) and between two epidermal cells (c). Vesicles probably directed to the periplasmic space (b) or to the vacuole (d). Patchy distribution of fluorescein (green) (e) and HPTS (green) (f) in epidermal cells of explant incubated in aqueous fluorochrome solution for 60 min (top surface views of the explant). Insets on (e) and (f) are orthogonal projections of samples depicted on corresponding figures proofing the presence of fluorochrome only in epidermal cells. TEM (a–d) and CLSM (e, f) images. Bars = 5 µm (a), 2 µm (b, c), 1 µm (d) and 100 µm (e, f). Am, amyloplast; C, cytoplasm; CW, cell wall; Cx, cortex; Ep, epidermis; G, electron-dense granules; ML, middle lamella; M, mitochondrion; N, nucleus; Nl, nucleolus; P, plastid; Pc, paracrystal bodies; ER, endoplasmic reticulum; V, vacuole; Vc, vesicles; arrowhead, point of fluorochrome application.

On the 6th day of culture, no epidermal cell of stipe explant had divided yet. Their cytoplasm was dense and rich in numerous organelles (Fig. 2a). The central part of the cell was occupied by the nucleus now. Their cell wall contained layers of electron-dense deposits and especially the middle lamella was strongly electron dense (Fig. 2b–d). In neighboring cortical cells, vesicles structurally resembling multivesicular bodies were present in the cytoplasm close to the vacuoles (Fig. 2d) or fused with plasmalemma indicating that unknown material is secreted into the periplasmic space (Fig. 2b).

Fluorescein was present only in some epidermal cells, thus giving a patchy pattern of fluorochrome distribution (Fig. 2e). Fluorescein was not detected in cortical cells (Fig. 2e, inset). A similar pattern of fluorochrome distribution was observed following HPTS treatment (Fig. 2f). These results indicate that the epidermis is no longer a single symplasmic domain. However, at this stage, it was not possible to determine which cells are symplasmically connected: the embryogenic or non-embryogenic.

### Fluorochrome distribution within one-dimensional linear somatic embryos (8th and 10th days of culture)

**Fig. 3:**
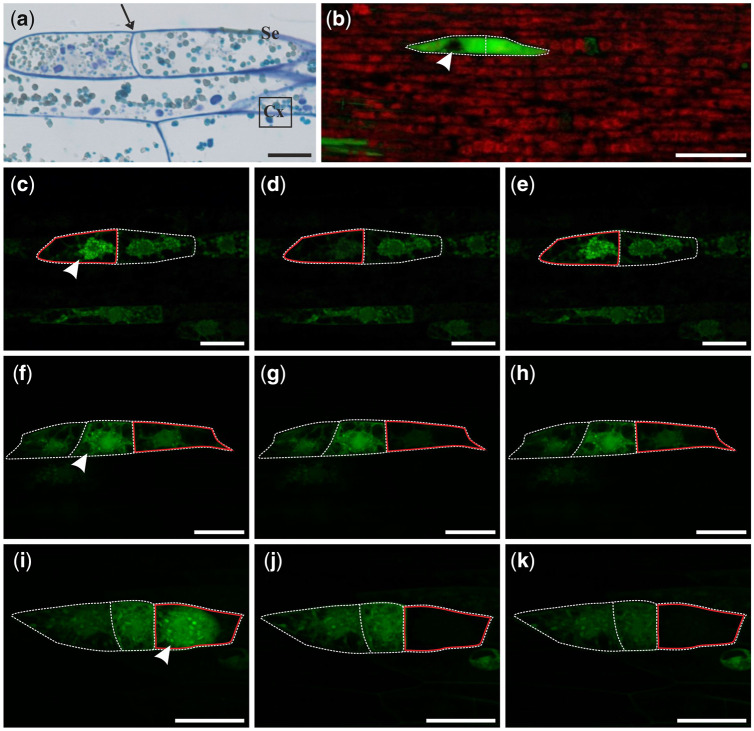
Histology and fluorochrome distribution in one-dimensional linear somatic embryo (Se) induced from single epidermal cells of stipe explants after 8 d (a–e) and 10 d of culture (f–k). (a) Longitudinal section of bicellular Se with well distinguishable cell wall between divided cells (arrow). (b) HPTS (green) accumulation in bicellular embryo and autofluorescence of chlorophyll (red) in adjacent epidermal cells. FRAP analysis conducted on bicellular (c–e) and tri-cellular embryos (f–k). Presence of fluorescein in embryo cells before FRAP (c, f, i), immediately after photobleaching (d, g, j), and after 5 min of recovery after photobleaching (e, h, k); top surface views of Se. Light microscopy after toluidine blue staining (a) and CLSM (b–k). Bars = 20 µm (a), 100 µm (b) and 50 µm (c–k). Se, somatic embryo; Cx, cortex; arrowhead, site of fluorochrome application; red line indicates the bleached cell; white dotted line indicates cell walls of examined cells of Se.

The first stage of somatic embryogenesis is a one-dimensional linear somatic embryo that is formed by the anticlinal divisions of a single epidermal cell (Fig. 3a). Somatic embryos at this stage are characterized by a specific cell arrangement, one by one, which results in their characteristic linear shape. Usually, embryos in this stage can be composed of up to 10 cells (Mikuła et al. 2015b).

At the 8th day of culture, when the epidermal cells start to divide (Fig. 3a), the flow of fluorochrome between dividing cells and non-dividing cells of the epidermis is stopped. HPTS provided to a single cell of the bicellular embryo moved to the second cell of the embryo but did not enter other cells of the explant (Fig. 3b, arrowhead points to the cell where fluorochrome was applied). This result was confirmed by FRAP technique (Fig. 3c–e). This experiment showed that, after photobleaching (Fig. 3d), the fluorescein moved to the bleached cell from the non-bleached cell (Fig. 3e). A similar process was usually observed in tri-cellular embryos (Fig. 3f–h), but at this stage, there were also embryos found where no movement of fluorescein between embryo cells took place after photobleaching (Fig. 3i–k). Both patterns of fluorochrome distribution were equally feasible as half of the examined embryos showed symplasmic coupling and the other half did not (Table 1). At the 10th day of culture, following the next set of anticlinal cell divisions, one-dimensional, multicellular linear embryos were formed (Table 1). HPTS distribution between the non-dividing epidermal cells of the explant and cells of the embryo was still limited (Table 1). These data show that a somatic embryo in an early stage of development (bicellular embryo) represents a single symplasmic domain for at least low-molecular-weight fluorochromes and does not exchange fluorochromes with neighboring explant cells. Moreover, it appears that, with an increase in the number of embryo cells, there is a reduction in fluorochrome exchange between them, which indicates that the symplasmic domain within the linear embryo has developed.

**Table 1:** Fluorochrome movement between somatic embryo (Se) and explant, and within Se

Developmental stage of Se	Explant → Se	Se → explant	Between embryo cells	
One-dimensional linear embryo				
2-Cell embryo 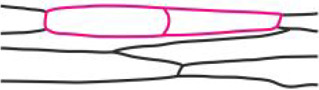	3^a^/8^b^	1/10	10/11	
3-Cell embryo 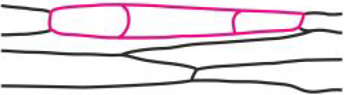	n/a	1/9	4/8	
6-Cell embryo 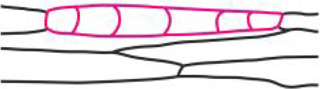	1/8	0/11	5/10	
Two-dimensional linear embryo 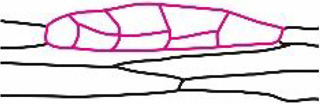	1/15	0/15	7/15	

			Within segment	Between segments

Spatial embryo 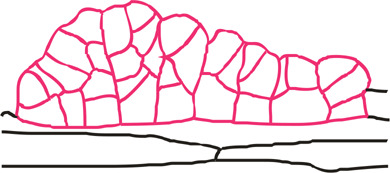	3/20	0/20	10/10	0/10

n/a, the cases that have not been analyzed.

a The number of cases when the fluorochrome is distributed between the analyzed areas.

b The number of analyzed cases.

### Fluorochrome distribution within two-dimensional linear somatic embryos (12th day of culture)

**Fig. 4:**
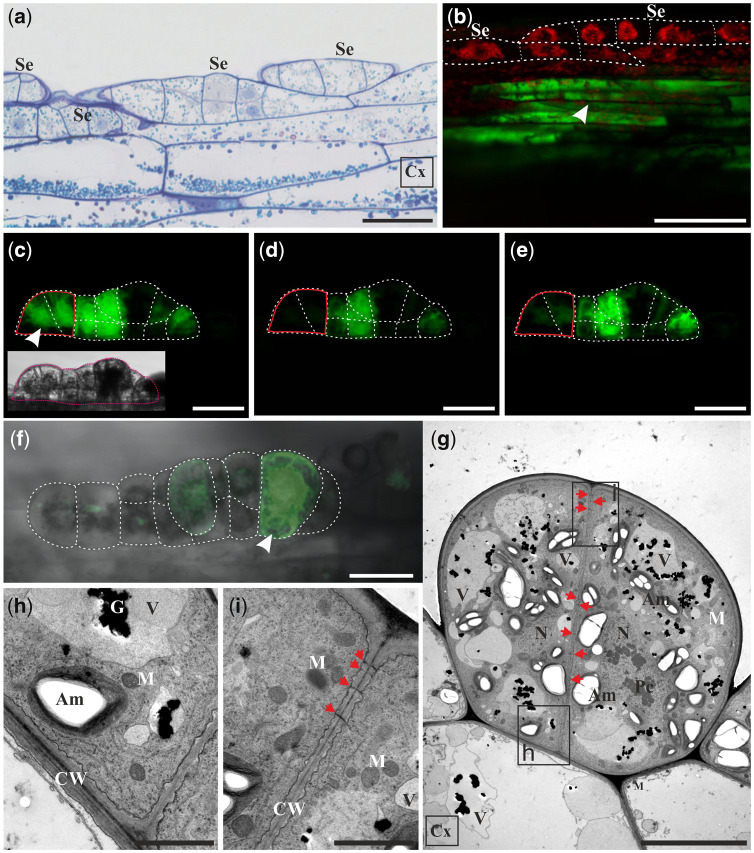
Microscopic analysis and fluorochrome distribution in two-dimensional linear somatic embryo (Se) at the 12th day of the culture. (a) Multicellular Se induced from single epidermal cells after a series of anticlinal and periclinal cell divisions (longitudinal section of tangent of the explant surface). (b) HPTS accumulation (green) occurs only in injured and neighboring epidermal cells of explant after incubation for 60 min in fluorochrome solution and is absent from Se cells. The red autofluorescence of chlorophyll in the cells of Se is only visible [embryos are at the same stage of development as embryos depicted in (a)]. (c–e) FRAP analysis (side surface view of Se). The area where fluorochrome was bleached is indicated by red line. Fluorescein fluorescence in the embryo before FRAP (c), immediately after photobleaching (d) and after 5 min of recovery after photobleaching (e). (f) Diverse distribution of fluorescein in the embryo body (top surface view of Se). (g–i) Ultrastructure of multicellular Se; the transverse section shows two embryo cells rich in cytoplasm and organelles connected with numerous plasmodesmata (red arrows). (h, i) Enlargements of areas marked with rectangles in (g). (h) Part of the cell wall between Se and explant. (i) Part of cell wall within the embryo. Light microscopy after toluidine blue staining (a), CLSM (b–f) and TEM (g–i). Bars = 20 µm (a), 100 µm (b), 50 µm (c–f), 10 µm (g) and 2 µm (h, i). Am, amyloplast; Cx, cortex; Se, somatic embryo; CW, cell wall; G, electron-dense granules; M, mitochondrion; Pc, paracrystal bodies; V, vacuole; red arrows, plasmodesmata; arrowhead, site of fluorochrome application; red line indicates the bleached cells. White dotted line indicates cell walls and outlines Se.

Following a series of anticlinal cell divisions, periclinal cell divisions of one-dimensional embryo began and the two-dimensional linear somatic embryo was formed (Fig. 4a). This somatic embryo has a linear shape consisted of a single layer of two-dimensionally arranged cells. As a result, the embryos protrude above the surface of the explant.

At the 12th day of culture, when HPTS was applied to epidermal cells of explants, it was not distributed to neighboring cells of two-dimensional embryos (Fig. 4b;Table 1). This clearly indicates that, at this stage of somatic embryo development, a symplasmic communication between explant cells and the embryo is restricted. FRAP analyses performed on the somatic embryo (Fig. 4c–e) showed that after the photobleaching of fluorescein in one part of the embryo (Fig. 4d), the fluorochrome moved to it from adjacent embryo cells (Fig. 4e). The distribution of fluorescein within somatic embryos was not uniform (Fig. 4c, f), and lack of fluorochrome in few cells was observed in several embryos examined. The results obtained suggest that somatic embryos are symplasmically isolated from explant cells and cell-to-cell communication via PD is restricted in both directions: from somatic cells to embryo cells and vice versa (Table 1). The cytoplasm of somatic embryo cells was rich in numerous small vacuoles and starch grains located in amyloplasts (Fig. 4g–i). The cell wall between somatic embryo cells and other cells of the explant consisted of layers that differed in electron density (Fig. 4h). The PD were found in cell walls between embryo cells (Fig. 4g, i).

### Fluorochrome distribution within spatial somatic embryos (16th day of culture)

**Fig. 5:**
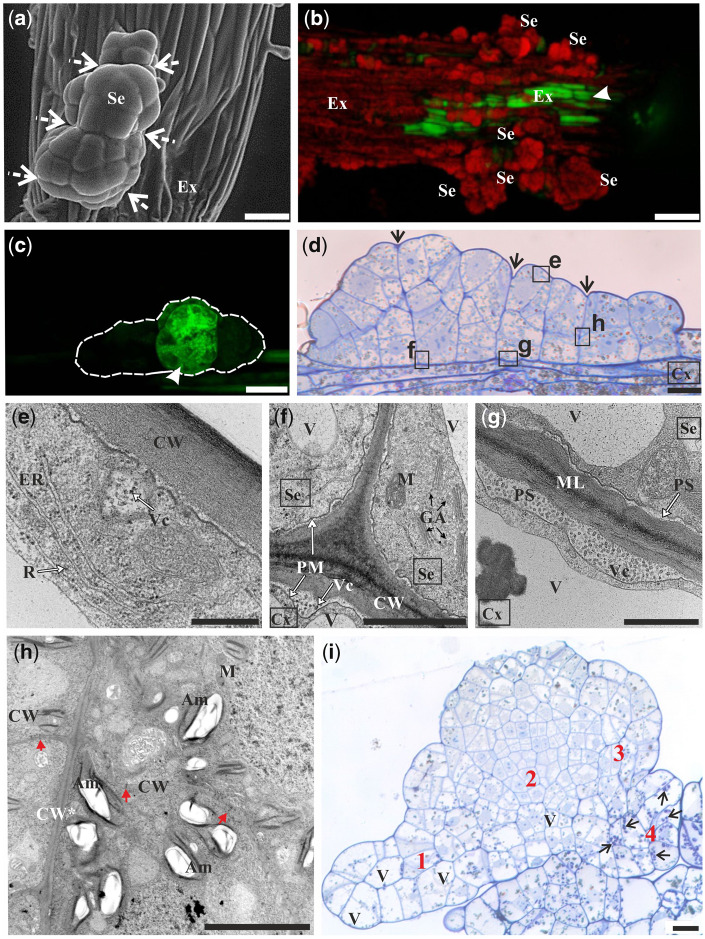
Microscopic analysis of spatial somatic embryo (Se) at the 16th day of the culture. (a) SEM image of the embryo with four segments (dashed arrows point to borders between segments). (b) HPTS accumulation (green) in some epidermal cells of explant near its application site (arrowhead) and autofluorescence of chlorophyll (red) in the cells of Se and other cells of the explant. (c) Localization of fluorescein is restricted to a specific segment of the embryo after fluorochrome application to the cell marked with an arrowhead (side surface view of Se). (d) Longitudinal section of Se composed of four segments. Arrows point to borders between segments. Selected areas [indicated by letters (e–h) next to squares] were analyzed by TEM. (e) Outer periclinal wall of the embryo and a fragment of cytoplasm with plastids, endoplasmic reticulum, ribosomes and vesicles. **(**f) Anticlinal wall between two cells of Se and inner periclinal wall between Se and cortex cell with well distinguishable middle lamella as an electron opaque layer. Undulated plasmalemma indicates intensive processes of exocytosis as confirmed by the presence of numerous dictyosomes of Golgi apparatus. (g) Deposition of numerous vesicles in the periplasmic space. (h) Cell wall between segments of Se. **(**i) Longitudinal section of Se composed of embryo regions with cellular heterogeneity: 1, large and vacuolated cells; 2, small and abundantly dividing cells; 3, medium-sized cells; 4, large cells being rich in starch (arrows). SEM (a), CLSM (b, c) light microscopy after toluidine blue staining (d, i) and TEM (e–h). Bars = 100 µm (a, c), 500 µm (b), 50 µm (d), 0.5 µm (e), 2 µm (f), 1 µm (g), 5 µm (h) and 20 µm (i). Am, amyloplast; CW, cell wall; CW*, cell wall between embryo segments; Cx, cortical cell; ER, endoplasmic reticulum; Ex, explant; GA, dictyosomes of Golgi apparatus; ML, middle lamella; M, mitochondrion; PM, plasma membrane; PS, periplasmic space; Se, somatic embryo; V, vacuole; Vc, vesicles; red arrows, plasmodesmata; arrowhead, site of fluorochrome application.

Further numerous anticlinal, periclinal and oblique cell divisions of the embryo body cells resulted in the formation of the spatial somatic embryo (Fig. 5a). This somatic embryo consisted of several layers of three-dimensionally arranged cells due to which its entire structure grows above the explant's surface horizontally and vertically.

After 16 d of culture, the embryo body consisted of four clearly delineated segments (Fig. 5a, d). HPTS distribution between the explant cells and somatic embryos was inhibited (Table 1). When fluorochrome was provided to cells of explant epidermis, it was never translocated to somatic embryos (Fig. 5b). Furthermore, when fluorochrome was applied to the somatic embryo, it did not move to explant cells (Fig. 5c). It indicates a lack of fluorochrome exchange between cells following different developmental programs at the late stage of somatic embryo development. In addition, restrictions in fluorescein movement between segments of the embryo were also clearly visible (Fig. 5c;Table 1). The somatic embryo, during segment differentiation, became isolated from the external environment (Fig. 5d, e) and from the explant cells (Fig. 5d, f) by a cell wall that was composed of layers differing in electron translucency. This stratification was visible for both embryo cells and cortical cells (Fig. 5g). Numerous vesicles were present in the periplasmic space of these cells. The PD were present between cells of the somatic embryos (Fig. 5h). The cytoplasm of somatic embryo cells was rich in endoplasmic reticulum structures, ribosomes, dictyosomes, mitochondria and small vacuoles (Fig. 5e, f, h). In developing somatic embryos, diversity in cell size, level of vacuolization and starch content were observed (Fig. 5i).

## Discussion

The formation of a complex multicellular organism from a single explant cell cultured under in vitro conditions is one of the most amazing processes in plant biology. The induction of this process demands activation of a new morphogenetic developmental program in explant cells. Some research indicates that changes in symplasmic communication may be of key importance for these processes (Ding et al. 1999, Marzec et al. 2014, Otero et al. 2016). In addition, research concerning SE is complicated due to often occurring indirect and side pathways of somatic embryo differentiation and multicellular origin of somatic embryos as sometimes several types of tissues may be involved. The model developed for the tree fern *C*. *delgadii* allows us to follow the transition of somatic cells into embryogenic ones. Furthermore, the subsequent formation of somatic embryo passing through the characteristic morphological stages, i.e. a series of anticlinal divisions and the formation of four segments of the embryo body, can be tracked down with high precision.

### Induction of SE

The studies presented here indicate that, during SE of the tree fern *C. delgadii*, a lack of exchange of substances that have similar physico-chemical properties to the fluorochromes used occurred. Thus, explant cells are symplasmically isolated during the acquisition of embryogenic competence.

In many works, plasmolysis is an effective factor inducing the changes in cell fate leading to an increase in the ratio of somatic embryo formation (Lou and Kako 1995, Choi and Soh 1997, Puigderrajols et al. 2001, Verdeil et al. 2001, You et al. 2006, Karami et al. 2006, Grimault et al. 2007). Plasmolysis disrupts the cellular interconnections between explant cells allowing more cells to undergo reprogramming and start to develop into somatic embryos. Published reports demonstrate that the exchange of information through PD may influence plant development and cell differentiation. Cells symplasmically connected by PD are characterized by a similar frequency and direction of divisions, and follow the same developmental program (Marzec and Kurczynska 2014). Spatio-temporal changes in symplasmic communication result in symplasmic domain formation in which the cells follow different developmental programs (Ehlers et al. 1999). The data presented here indicate that in the case of fern SE, relationships between symplasmic isolation and changes in cell fate, including reestablishment of cellular totipotency, also occur. These results are new for fern SE and are consistent with data obtained from angiosperm plants (Kulinska-Lukaszek and Kurczynska 2012, Kurczynska et al. 2007). Thus, our results seem to confirm that the interruption of cell-to-cell communication stimulates the reprogramming of somatic cells into an embryogenic state and induces SE. At present, we are not able to explain the mechanism of this interruption. However, we assume that it may be based on a series of physiological rearrangements that the explant undergoes at an early stage before the cell divisions leading to somatic embryo formation occur (Grzyb et al. 2017). Among them, a sudden increase in the concentration of soluble sucrose is one of the first and the most discernible reactions that may switch on the developmental program, which allows certain epidermal cells to regain their potential for SE.

### Somatic embryo development

During the development of somatic embryos in *C. delgadii* , there is a temporary loss of communication between cells of the embryo body. Changes in PD permeability appear first at the tri-cellular embryo stage. An interruption of cell-to-cell communication does not interfere with the occurrence of further divisions, which are only transverse to the axis of the explant. After 8–10 divisions, restrictions in symplasmic communication are still present in the embryo. At this point, reduced cytoplasmic flow can lead to the divisions occurring in a different orientation and with variable frequency. Contrary to our results, in the early zygotic embryo of Arabidopsis, all cells form a single symplasmic domain until the early torpedo stage (Kim et al. 2005).

Changes in the direction of cellular divisions in the one-dimensional linear somatic embryo of *C*. *delgadii*, as well as frequency of divisions, lead to the formation of four segments, each being separate symplasmic subdomain. Our study showed that symplasmic communication is restricted between the embryo segments, but cells of the same segment are symplasmically interconnected. Similarly, Kim et al. (2002) showed that the occurrence of symplasmic domains in the zygotic embryo of Arabidopsis precedes the differentiation of organs i.e. roots and leaves. However, only transport of large-molecular-weight tracer (10 kDa fluorescent dextran) was limited, whereas the smaller-molecular-weight tracer (HPTS) was transported in all stages of embryogenesis. We speculate that symplasmic isolation is extremely important in the process of differentiation into four segments of the somatic embryo in ferns. Although at present we cannot yet indicate which organ becomes differentiated from each segment of the *C. delgadii* embryo, we can only assume that the most actively dividing segment (such as region 2 in Fig. 5i) will give rise to the first leaf. Our previous studies showed that this segment of the zygotic embryo of *C. delgadii* develops faster than the others (Mikuła et al. 2018). It seems that the difference in the intensity of cellular divisions may result from the unequal transport of hormones and other molecules or signals between particular segments of the embryo body. In plant embryogenesis, positional information establishes the overall body plan and lineage-dependent cell fate specifies local patterning. Taking Arabidopsis as a model, researchers emphasize that auxin signaling (Berleth and Chatfield 2002) in combination with specific genes products (Laux et al. 2004) regulates the formation of the basic apical–basal body pattern in early embryos. Moreover, the movement of auxin through PD was elucidated by studies of the Arabidopsis mutant *gsl8*, which has increased PD permeability (Han et al. 2014). Thus, our studies show PD as dynamic intercellular channels for the transport of embryo-specific factors regulating somatic embryo development.

Considering that somatic embryo cells become symplasmically isolated from explants early upon their induction, and the isolation persists at least until the embryo reaches the spatial stage, the question remains of how the embryo is nourished. Bell (1986) showed that starch is accumulated abundantly in the egg cells of a number of ferns. In accordance, our studies revealed the numerous starch grains accumulating in some cells of the explant epidermis, and also in the somatic embryos composed of several cells. This may provide an energy reserve allowing somatic embryo development in the absence of symplasmic connection with the explant. In young zygotic embryo, the foot expands and differentiates to facilitate the transport of nutrients from the gametophyte to the developing embryonic organs (Johnson and Renzaglia 2008). Although some parts of the four-segment somatic embryo of *C. delgadii* contain more starch deposits than others, we were not able to document the development of placenta.

## Conclusions

1. The somatic-to-embryogenic transition is preceded by restrictions in the distribution of low-molecular-weight fluorochromes between cells of the initial explant. Thus, we assume that symplasmic isolation of selected explant cells is a prerequisite for reprogramming of cell development and initiation of the morphogenetic response.
2. The bicellular somatic embryo is a single symplasmic domain.
3. The restrictions in the fluorochrome flow appear first in tri-cellular embryos and the embryo ceases to be a single symplasmic domain.
4. In fern somatic embryo, symplasmic subdomains are formed that correspond to the four segments of the embryo body. Thus, we conclude that changes in the PD connections are prerequisite for regular embryo development.
5. The data presented provide a novel view of fern SE as a process regulated by symplasmic communication.

## Materials and Methods

### Plant material and culture conditions

Sporophytes that had developed two or three leaves were used as a source of explants. Plant material was collected from the youngest fronds of 5-month-old in vitro-grown sporophytes maintained in darkness. The plant material was cultured on a hormone-free agar medium containing half-strength macro- and micronutrients basal MS medium (Murashige and Skoog 1962) supplemented with vitamins and 1% sucrose (Mikuła et al. 2015a).

To carry out microscopic analyzes, the plant material was successively collected after 6, 8, 10, 12 and 16 d of culture. Stipe explants, about 2.5 mm in length, were freshly cut off from the first fronds of donor sporophytes and used as initial explants. Five to twenty explants were analyzed for each time point.

### Fluorescent probes

Symplasmic communication was investigated using low-molecular-weight fluorochromes: 8-hydroxypyrene-1,3,6-trisulfonic acid, trisodium salt (HPTS; Sigma-Aldrich, St. Louis. Missouri, USA; H1529) and fluorescein. HPTS is a membrane impermeable fluorochrome, and a recommended symplasmic tracer (Wright and Oparka 1996). Thus, the plasma membrane was injured with a microcapillary to allow its uptake into cytoplasm (see below). Fluorescein was delivered to cells in the form of FDA (Sigma-Aldrich, F7378). FDA is a nonfluorescent, membrane-permeant compound. After uptake into the cytoplasm, cell esterases cleave off acetate groups and release fluorescein into the cytoplasm. Fluorescein is fluorescent and membrane impermeant (Wright and Oparka 1996). In experiments, HPTS fluorescence was more stable than fluorescein fluorescence, so it could be observed for a long time without bleaching. As fluorescein was very sensitive for bleaching it was chosen for FRAP experiments (fluorescence recovery after photobleaching; described below). Both fluorochromes (HPTS and fluorescein) upon uptake into cytoplasm move from one cell to another only via PD and, thus, were used in the studies of symplasmic communication (Gisel et al. 2002, Wrobel et al. 2011, Maule et al. 2013, Milewska-Hendel et al. 2019). For symplasmic communication analyses, HPTS solution (5 mg/ml in water) and FDA solution (5 mg/ml stock FDA in acetone diluted 1:10 in water) were used.

### Application of fluorochromes

**Fig. 6:**
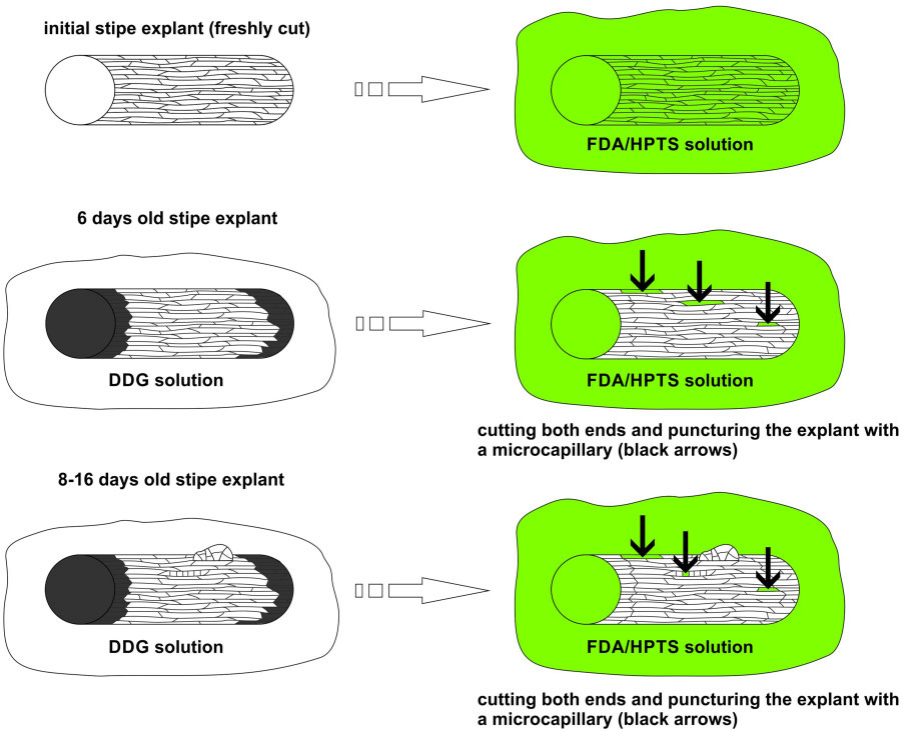
Scheme of fluorochromes application to initial stipe explants and to explants cultured for 6–16 d. HPTS, 8-hydroxypyrene-1,3,6-trisulfonic acid, trisodium salt; marked in black, two ends of explant browning during the culture; marked in green, fluorochrome solution.

Analysis of symplasmic communication was conducted on initial explants and explants cultured for 6–16 d (Fig. 6). The initial explants were incubated for 60 min in HPTS (or FDA) solution immediately after excision from the sporophyte. Cultured explants were incubated in 0.1 mM aqueous solution of 2-deoxy-d-glucose (DDG; Sigma-Aldrich, D6134) for 30–60 min to prevent callose deposition. Explants were then immersed in HPTS (or FDA) solution and punctured randomly (by hand, under a stereomicroscope) with a microcapillary of outer diameter between 7 and 10 µm. Subsequently, both ends of explants were cut off and explants were incubated in the fluorochrome solution for 60 min. After incubation, the plant material was rinsed with demineralized water to wash out the excess of fluorochrome and then analyzed under a confocal laser scanning microscope (CLSM) as described below.

### Viability tests

Cell viability tests were performed with 0.01 µg/ml solution of propidium iodide (PI; Sigma-Aldrich, P4170) and 0.01% (w/v) solution of Evans blue (Sigma-Aldrich, E2129) dyes. Plant material was incubated in PI solution after FDA (or HPTS) treatment. Thus, it was possible to collect images of fluorescein (or HPTS) and PI simultaneously (see below). Explants were stained with Evans blue solution after CLSM analysis, because this dye is used for bright field microscopy and its hue is not visible in the ‘bright field’ mode of CLSM. Dead cells in explants were not analyzed.

### Confocal laser scanning microscopy

For direct, noninvasive, serial optical sectioning of intact, thick, living samples, a confocal laser scanning microscope FV 1000 (Olympus, Shinjuku, Tokyo, Japan), which is built onto IX81 inverted fluorescence microscope equipped with a multi-band argon laser, was used to excite fluorescence of fluorochromes. HPTS and fluorescein were excited with 488 nm wavelength, and emission was collected at 500–530 nm. In all analyses, the laser power was set at 5–10% for HPTS excitation and 2–4% for fluorescein excitation. The voltage on photomultipliers (PMTs) was set at 530–700 V for both fluorochromes. PI was excited with 543 nm wavelength, and emission was collected at 555–625 nm (laser power: 23–25%, PMT: 680–700 V).

FRAP experiments were performed on plant material incubated in FDA. First, the region of interest was selected using CLSM software (FluoView 1000) and it was irradiated with 405 nm laser (laser power set at 60–90%) for 30–60 s to bleach the fluorescein. Next, time-series images were collected to check whether the fluorochrome had returned to the bleached area/cells (excitation 488 nm). The mean value of fluorescein fluorescence was measured in the bleached area just after bleaching and during the next 3–5 min. If the mean value of fluorescence rose by 20% (or more), it was assumed that the recovery occurred. Time-series analyses were carried out for 3–5 min because the prolonged acquisition of images resulted in a reduction in fluorescein fluorescence intensity.

Before collecting data, autofluorescence of explants and embryo cells was checked for all plant material. For analyses of symplasmic fluorochrome movement, CLSM settings were used when no autofluorescence signal was collected by PMTs in control unstained samples, with an exception of chlorophyll autofluorescence. However, emission wavelengths of chlorophyll autofluorescence (630–700 nm) did not overlap with those of HPTS and fluorescein (500–530 nm) and chlorophyll autofluorescence could be examined simultaneously with both fluorochromes.

Digital CLSM images were prepared for publication using FluoView and ImageJ software. Data for fluorochrome movement between explant and somatic embryo cells, and within somatic embryo segments, are given in Table 1.

### Transmission electron microscopy, scanning electron microscopy and light microscopy analyses

Plant material was prepared as described previously (Domżalska et al. 2017). The explants were fixed in a mixture of 2.5% (w/v) paraformaldehyde and 2.5% (v/v) glutaraldehyde in 0.05 M sodium cacodylate buffer (pH 7.2; room temperature; 24 h). Next, the samples were post-fixed in 2% osmium tetroxide in 0.05 M cacodylate buffer at 4°C for 6 h and dehydrated in a graded ethanol series (30, 50, 70, 90, 96 and 3 times 99.8%) for 2 h at each concentration, followed by propylene oxide substitution. The samples were infiltrated with graded series of Epon epoxy resin (Sigma-Aldrich) mixtures for 48 h in total and polymerized at 65°C for 16 h. For transmission electron microscopy (TEM), ultrathin sections (90-nm thick) were cut with a Leica EM UC6 ultramicrotome (Leica, Wetzlar, Germany) and collected onto carbon-coated copper grids (300 mesh). They were contrasted in a saturated solution of uranyl acetate dissolved in 50% (w/v) ethanol (30 min) and 0.04% (w/v) lead citrate (30 min). Specimens were examined with an FEI 268D ‘Morgagni’ (FEI Corp., Akishima, Hillsboro, USA) transmission electron microscope operating at 80 kV equipped with an Olympus-SIS ‘Morada’ 11 Mpix digital camera or with a Jeol JEM-3010 (Jeol, Tokyo, Japan) high-resolution electron microscope (accelerating voltage 300 kV) equipped with a Gatan 2k × 2k Orius TM 833 SC200D CCD camera. For TEM analysis, at least 20 sections through all tissue layers of the explant were examined.

For light microscopy, 2-μm-thick sections were collected onto microscope slides. Samples were stained with 0.1% (w/v) toluidine blue in 1% (w/v) borax. The semi-thin sections were observed using a light Olympus Vanox microscope.

For scanning electron microscopy (SEM) analyses, non-fixed plant material was examined with the FEI Quanta 200 environmental scanning electron microscope at a relative humidity of up to 100% and at a vacuum of <10^−4 ^Pa.

## Funding

Polish National Science Centre (NCN) [Grant No. 2017/27/N/NZ3/00434].
